# Melanoma in a patient with DNMT3A overgrowth syndrome

**DOI:** 10.1101/mcs.a006267

**Published:** 2023-04

**Authors:** David Y. Chen, Leslie A. Sutton, Sai Mukund Ramakrishnan, Eric J. Duncavage, Sharon E. Heath, Leigh A. Compton, Christopher A. Miller, Timothy J. Ley

**Affiliations:** 1 Division of Dermatology, Washington University School of Medicine, St. Louis, Missouri 63110, USA;; 2 Division of Oncology, Washington University School of Medicine, St. Louis, Missouri 63110, USA;; 3 Department of Pathology and Immunology, Washington University School of Medicine, St. Louis, Missouri 63110, USA

**Keywords:** cutaneous melanoma, overgrowth

## Abstract

Alterations in epigenetic regulators are increasingly recognized as early events in tumorigenesis; thus, patients with acquired or inherited variants in epigenetic regulators may be at increased risk for developing multiple types of cancer. DNMT3A overgrowth syndrome (DOS), caused by germline pathogenic variants in the DNA methyltransferase gene *DNMT3A*, has been associated with a predisposition toward development of hematopoietic and neuronal malignancies. DNMT3A deficiency has been described to promote keratinocyte proliferation in mice. Although altered DNA methylation patterns are well-recognized in melanoma, the role of DNA methyltransferases in melanoma pathogenesis is not clear. We report the case of an adult DOS patient with a germline *DNMT3A* loss-of-function mutation, who developed an early-onset melanoma with regional lymph node metastatic disease. Exome sequencing of the primary tumor identified an additional acquired, missense *DNMT3A* mutation in the dominant tumor clone, suggesting that the loss of DNMT3A function was relevant for the development of this tumor.

## INTRODUCTION

DNA methylation is an epigenetic process that is established by the de novo DNA methyltransferases DNMT3A and DNMT3B, and maintained by DNMT1 (Li et al. 1992; Okano et al. 1998). DNMT3A overgrowth syndrome (MIM #615879) consists of a constellation of clinical manifestations with three principal features, including overgrowth (tall stature, increased head circumference, elevated body mass index), impaired intellectual development, and characteristic facial features, and is associated with de novo germline heterozygous mutations in the DNA methyltransferase gene *DNMT3A* (Tatton-Brown et al. 2014). Since its first description, the number of patients with documented DNMT3A overgrowth syndrome (DOS) has grown to more than 300 at present, whereas the repertoire of clinical manifestations has expanded to include obesity, cardiac defects, umbilical hernia, hypotonia, joint hypermobility, seizures, behavioral disorders, and other phenotypes (Hollink et al. 2017; Kosaki et al. 2017; Shen et al. 2017; Xin et al. 2017; Tatton-Brown et al. 2018; Jeffries et al. 2019; Balci et al. 2020; Ferris et al. 2021; Smith et al. 2021; Cecchi et al. 2022).

In addition to its syndromic features, DOS patients are at higher risk for certain malignancies, including hematologic malignancies (Hollink et al. 2017; Smith et al. 2021). Clonal hematopoiesis is caused most frequently by somatic mutations in *DNMT3A*, which are also a common feature of normal karyotype acute myeloid leukemia (AML) (Ley et al. 2010, 2013; Jaiswal et al. 2014; Xie et al. 2014; Genovese et al. 2015). Protein altering mutations at arginine 882 typically confer dominant-negative DNA methyltransferase activity, resulting in focal, canonical DNA hypomethylation patterns in DOS patient blood cells and in AML cells (Ley et al. 2010; Kim et al. 2013; Russler-Germain et al. 2014; Smith et al. 2021). DNMT3A haploinsufficiency has been shown to lead to a milder DNA hypomethylation phenotype, and corresponding preclinical models are predisposed to develop spontaneous and oncogene-induced leukemia (Meyer et al. 2016; Cole et al. 2017). Although other tumor types have been described in DOS patients, skin cancer has not yet been reported. We present the first known case of a DOS patient who developed early-onset melanoma, associated with a second acquired *DNMT3A* mutation, suggesting a role for loss-of-function *DNMT3A* mutations in melanoma initiation.

## RESULTS

### Clinical Presentation and Family History

**Figure 1.**
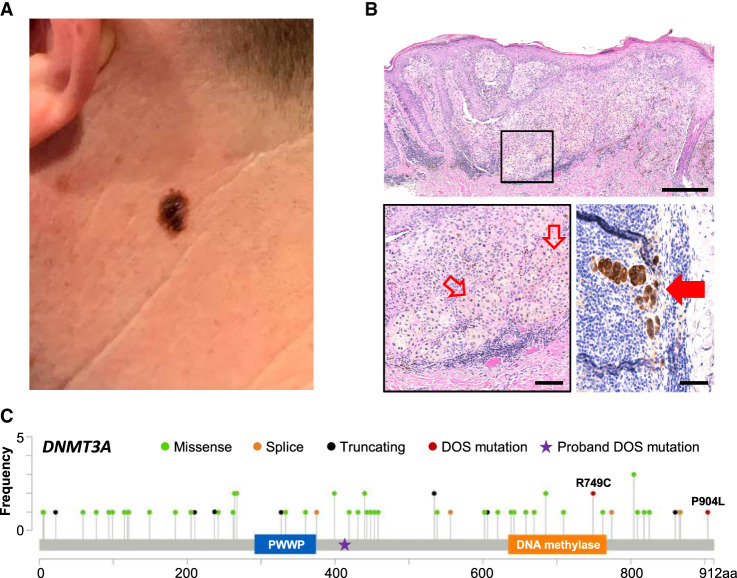
Invasive melanoma in a DNMT3A overgrowth syndrome (DOS) patient. (*A*) Clinical appearance of a thin plaque with irregular borders and heterogeneous pigmentation on the left lateral neck. (*B*) Hematoxylin and eosin (H&E)-stained (*top*) punch biopsy specimen consisting of atypical melanocytes with at the dermal–epidermal junction, as well as pagetoid spread and an invasive dermal component, measuring 1.3 mm in thickness (scale = 250 µm) with higher magnification of the invasive component with a mitotic cell highlighted by hollow red arrows (scale = 100 µm; boxed *inset* and *lower left* panel) and MART1 stain highlighting metastatic melanoma (solid red arrow) in the sentinel lymph node (scale = 50 µm; *bottom right*). Sequencing was performed on the other half of this bisected specimen. (*C*) *DNMT3A* variants in melanoma curated from cBioPortal (Cerami et al. 2012; Gao et al. 2013). Proband F414fs* germline mutation is notated with a star, whereas the acquired *DNMT3A*^R749C^ melanoma mutation is additionally a mutation previously described in DOS patients.

The proband is a male diagnosed with *DNMT3A* overgrowth syndrome, with a de novo heterozygous insertion in the *DNMT3A* gene (c.1238dupG) resulting in DNMT3A^F414fs*^; peripheral blood cells from this patient had been analyzed in a prior report demonstrating a haploinsufficiency phenotype for DNA methylation (Smith et al. 2021). He is the second child of four, and his three siblings are unaffected. He has mild developmental delay and attention deficit hyperactivity disorder, as well as aortic root dilatation. At 20 yr of age, he developed a dermatofibrosarcoma protuberans on the lower back that was surgically resected. At 34 yr of age, he presented with a growing, pigmented skin lesion on his left neck (Fig. 1A). A punch biopsy through one part of the lesion revealed a compound melanocytic proliferation composed of severely atypical epithelioid cells arranged as single cells and nests at all levels of the epidermis and as dermal nests that lack maturation with tumor depth. Ulceration is not present, and mitoses number up to 2/mm^2^. These findings are compatible with malignant melanoma, invasive to a thickness of 1.3 mm (Fig. 1B, top and bottom left panels). Additional histologic levels reveal an associated nevus. Wide local excision with sentinel lymph node biopsy revealed metastatic melanoma in two out of three lymph nodes sampled in the left parotid tail without extracapsular extension (Fig. 1B, bottom right panel), whereas positron emission tomography-computed tomography (PET-CT) and brain magnetic resonance imaging (MRI) were negative for overt metastatic disease, consistent with stage IIIA (pT2aN2aM0) disease. He then received adjuvant immune checkpoint blockade with pembrolizumab and has more than 8 mo of follow-up without evidence of recurrent or metastatic disease.

### Genomic Analyses

We performed whole-exome sequencing using genomic DNA derived from the biopsy sample of the primary melanoma, which redemonstrated the patient's constitutional *DNMT3A*c.1238dupG mutation with a variant allele frequency (VAF) of 0.5. Tumor-specific, somatically acquired variants were defined by comparison to a previously obtained whole-exome data set from the peripheral blood of the same patient several years before (Smith et al. 2021). This analysis identified an acquired *BRAF*c.1799_1800delinsAC mutation corresponding to the BRAF^V600D^, which is rarely found in melanoma (0.4%; Greaves et al. 2013) compared to the canonical melanoma oncogene BRAF^V600E^, found in >50% of cutaneous melanomas (Cancer Genome Atlas Network et al. 2015). Importantly, we also detected an acquired missense variant in *DNMT3A*c.2245C > T causing an arginine to cysteine change at amino acid 749 (R749C), which is known to cause methyltransferase loss-of-function (Christian et al. 2020) and which is associated with DOS (Tatton-Brown et al. 2018). The *BRAF*^V600D^ and *DNMT3A*^R749C^ mutations both had a VAF of 0.12, suggesting that 24% of the cells in the sample contained these two mutations; this frequency is consistent with the tumor burden in this sample (∼25% of total cells, see Fig. 1B), suggesting that both mutations were in the dominant melanoma clone in the skin lesion. *NF1* and *NRAS* did not harbor somatic mutations. Interestingly, we also detected a somatic *GNAQ*^R183Q^ mutation (previously described in uveal melanomas) in the dominant clone (Table 1; Robertson et al. 2017).

**Table 1.** Selected melanoma tumor somatic invariants identified by exome sequencing

Gene	Chromosome	VAF	HGVS DNA reference	HGVS protein reference	Variant type	Predicted effect
*BRAF*	7	0.124	ENST00000646891.1:c.1799_1800delinsAC	ENSP00000493543.1:p.Val600Asp	Deletion-insertion	Missense
*DNMT3A*	2	0.121	ENST00000264709.7:c.2245C>T	ENSP00000264709.3:p.Arg749Cys	Substitution	Missense
*GNAQ*	9	0.163	ENST00000286548.8:c.548G>A	ENSP00000286548.4:p.Arg183Gln	Substitution	Missense

(VAF) Variant allele frequency, (HGVS) Human Genome Variation Society.

## DISCUSSION

Multiple cancers exhibit altered DNA methylation patterns, typified by global DNA hypomethylation and focal hypermethylation (Jones and Baylin 2007; Ehrlich 2009). In melanoma, DNA methylation is thought to play a complex role in regulating the expression of canonical tumor suppressor genes, as well as genes that modulate homeostatic processes including cellular proliferation, metabolism, and immune response (Micevic et al. 2017). However, the contributions of individual DNA methyltransferases to skin cancer pathogenesis are incompletely understood. In preclinical studies, Dnmt3b deficiency was shown to prevent melanoma development in Braf- and Pten-deficient murine melanocytes through modulation of the mTOR pathway (Micevic et al. 2016). Studies in the B16 murine melanoma line, on the other hand, suggested a requirement for functional Dnmt3a in melanoma survival and proliferative capacity (Deng et al. 2009; Kim et al. 2018a).

In human melanomas, somatic *DNMT3A* variants have been detected in 3.5% (65/1849) of the sequenced melanoma samples in cBioportal (Berger et al. 2012; Cerami et al. 2012; Hodis et al. 2012; Krauthammer et al. 2012; Gao et al. 2013; Snyder et al. 2014; Van Allen et al. 2014, 2015; Cancer Genome Atlas Network et al. 2015; Hugo et al. 2016; Catalanotti et al. 2017; Liu et al. 2019; Shoushtari et al. 2021), which are distributed throughout the gene; they include the R749C and P904L variants described in DOS patients (Fig. 1C; Tatton-Brown et al. 2018), as well as multiple nonsense mutations predicted to cause early termination prior to the methyltransferase domain. In the present case, the proband has a germline, truncated, loss-of-function mutation in *DNMT3A*. Methylation analysis of his peripheral blood cells revealed a hypomethylation phenotype that was consistent with that of DNMT3A haploinsufficiency (Smith et al. 2021). The acquired missense *DNMT3A* mutation in the dominant clone (which is also known to cause loss of function) suggests a strong selective pressure for near-total DNMT3A loss of function that probably represented the initiating event for this tumor. Interestingly, there was a notable absence of the canonical melanoma *BRAF*/*NRAS*/*NF1* driver mutations; instead, we detected a noncanonical *BRAF*^V600D^ mutation that, although rare in melanoma (0.4% of cases), retains sensitivity to dabrafenib kinase inhibition in cell lines, implicating its role as an oncogenic driver (Gentilcore et al. 2013; Greaves et al. 2013). In the present case, we also identified a *GNAQ*^R138Q^ variant that has been previously described in uveal melanoma and that exhibits activation of the MAPK pathway in vitro, although not as strongly as the more common *GNAQ*^R209^ variants (Shirley et al. 2013). The G protein α-subunit *GNAQ* is mutated in 46% of uveal melanomas and 84% of blue nevi samples (van Raamsdonk et al. 2009) and is less frequently (2.4%) altered in cutaneous melanoma, suggesting that DNMT3A loss of function may be more impactful for melanoma development in certain contexts.

Most cases of DOS-associated malignancies have been described in the pediatric and young adult populations (Hollink et al. 2017; Tatton-Brown et al. 2018; Ferris et al. 2021). Melanoma is typically a disease diagnosed in later life, with a median age of 65 yr at diagnosis (SEER21). Melanoma in males in their fourth decade accounted for 2.8% of all cases of invasive melanomas diagnosed between 2001 and 2015; in this limited group, only a small fraction (6.2%) of patients exhibited metastatic disease to regional lymph nodes (Paulson et al. 2020). It is intriguing that the proband had previously developed a rare skin cancer at age 25—dermatofibrosarcoma protuberans—a cutaneous sarcoma with an estimated incidence of 4.2 cases per million in the United States, and a peak incidence in the fourth and fifth decades of life (Criscione and Weinstock 2007). Although the development of two uncommon cancers is not direct evidence that DNMT3A haploinsufficiency generates a premalignant state for skin tumors, preclinical models have suggested that Dnmt3a deficiency causes proliferative priming as a potential mechanism (Rinaldi et al. 2017; Chen et al. 2021). These findings suggest that dermatologic surveillance and preventative measures (counseling UV protection), may be an important part of comprehensive care for patients with DOS.

Finally, an important consideration in DOS and other constitutive disorders is that the molecular alterations that promote tumorigenesis in these patients may also have systemic implications that have a bearing on therapeutic response. There are two adjuvant therapies currently in use for resected stage III melanoma—immunotherapy versus targeted therapy for *BRAF*^V600E^-mutated melanoma (Long et al. 2017; Eggermont et al. 2018). Preclinical studies demonstrate that Dnmt3a-dependent de novo DNA methylation reduces the efficacy of PD1 checkpoint blockade by promoting CD8 T-cell exhaustion, whereas Dnmt3a inhibition can aid in T-cell rejuvenation (Ghoneim et al. 2017). Although the role of checkpoint blockade in tumors occurring in DOS patients is currently is unknown, DNMT3A haploinsufficiency could potentially affect its efficacy.

## METHODS

### Genome Sequencing and Data Analysis

A tissue core was obtained from paraffin-embedded tissue from the primary melanoma tumor biopsy specimen, using a 1-mm punch biopsy tool. Genomic DNA was extracted, and sequencing libraries were generated with IDT xGen exome kit version 1, and then sequenced on the Illumina Novaseq 6000 platform using an S4 flow cell. Sequence data was aligned against reference sequence hg38 using BWA-MEM (Li 2013). The aligned reads were sorted, deduplicated, and run through base quality score recalibration (BQSR). Sequence variants were called against reference blood from this patient, which was sequenced in a prior report (UPN 228211; Smith et al. 2021). Structural variants (SVs) and large indels were detected using Manta (Chen et al. 2016). Single-nucleotide variants (SNVs) and small indels were detected using VarScan2 (Koboldt et al. 2012), Strelka2 (Kim et al. 2018b), and MuTect2 (Cibulskis et al. 2013). Variants with a population frequency in gnomAD of >0.1% were removed, as were those in regions of low-quality mapping and low coverage (<20×). Variant annotation was performed with the Variant Effect Predictor, version 95 (McLaren et al. 2016). The entire somatic pipeline is available as a CWL workflow at https://github.com/genome/analysis-workflows (CommitID: 3e653e78fea91cf9c487534ceca1db328b6b68e0; commitURL: https://github.com/genome/analysis-workflows/commit/3e653e78fea91cf9c487534ceca1db328b6b68e0).

## ADDITIONAL INFORMATION

### Data Deposition and Access

Sequencing data for this patient was deposited to dbGaP (phs000159). Tha variants were submitted to ClinVar (https://www.ncbi.nlm.nih.gov/clinvar/) and can be found under accession numbers SCV003914810–SCV003914813 for NM_022552.5:c.1238dupG, NM_004333.6:c.1799_1800delinsAC, NM_022552.5:c.2245C>T, and NM_002072.4:c.548G>A, respectively.

### Ethics Statement

Patient data, samples, and photos were collected and analyzed with written patient consent under the IRB approved protocol #201011766, which explicitly allows for potentially identifying genomic studies, including genome sequencing, and approved by the Human Research Protection Office at Washington University School of Medicine.

### Acknowledgments

We thank the patient and his family for contributing to this study and his physician Michael E. Gifford, MD for procuring the tumor tissue sample in this study.

### Author Contributions

T.J.L. conceptualized the project, D.Y.C. and T.J.L. wrote the primary draft, L.A.S., S.M.R., C.A.M., and D.Y.C. performed the formal analysis, E.J.D., S.E.H., and L.A.C. provided clinical support, and D.Y.C., L.A.S., S.M.R., E.J.D., C.A.M., and T.J.L. edited the article.

### Funding

This study was supported by grants from the National Institutes of Health CA237727 (to D.Y.C.), CA211782 (to C.A.M.), and CA101937 and CA197561 (to T.J.L.) and from the Barnes Jewish Foundation (to T.J.L.).

### Competing Interest Statement

The authors have declared no competing interest.
